# Differences in HIV risk factors between South African adolescents and adult women and their association with sexually transmitted infections

**DOI:** 10.1136/sextrans-2024-056260

**Published:** 2024-12-04

**Authors:** Pamela Mkhize, Celia Mehou-Loko, Nokuthula Maphumulo, Nina Radzey, Andrea G Abrahams, Sengeziwe Sibeko, Rushil Harryparsad, Monalisa Manhanzva, Bahiah Meyer, Phumla Radebe, Lenine J P Liebenberg, Sinaye Ngcapu, Nadia Ahmed, Funeka Busakwe, Noluthando Mqaba, Derseree Archary, Aida Sivro, Natasha Samsunder, Disebo Potloane, William Horsnell, Christine Jordan, Quarraisha Abdool Karim, Linda-Gail Bekker, Jo-Ann Passmore, Heather Jaspan, Hilton Humphries, Lindi Masson

**Affiliations:** 1 Centre for the Aids Programme of Research in South Africa, Durban, South Africa; 2 Biochemistry Department, University of KwaZulu-Natal, Pietermaritzburg, South Africa; 3 Department of Pathology, University of Cape Town, Cape Town, South Africa; 4 Human Sciences Research Council, Pretoria, South Africa; 5 Stellenbosch University, Stellenbosch, South Africa; 6 Department of Medical Microbiology, University of KwaZulu-Natal, Durban, South Africa; 7 Sexual Health and HIV, Central and North West London NHS Trust, London, UK; 8 University of Cape Town Desmond Tutu HIV Centre, Cape Town, South Africa; 9 JC Wilt Infectious Disease Research Centre, National Microbiology Laboratory, Public Health Agency of Canada, Winnipeg, Manitoba, Canada; 10 Department of Medical Microbiology and Infectious Diseases, University of Manitoba, Winnipeg, Manitoba, Canada; 11 Medical Research Council Centre for Medical Mycology, University of Exeter, Exeter, UK; 12 Institute of Infectious Diseases and Molecular Medicine, University of Cape Town, Cape Town, South Africa; 13 Life Sciences Discipline, Burnet Institute, Melbourne, Victoria, Australia; 14 National Health Laboratory Service, Johannesburg, South Africa; 15 Seattle Children's Hospital, Seattle, Washington, USA; 16 Centre for Community-Based Research, Human Sciences Research Council, Pretoria, South Africa; 17 Department of Psychology, University of KwaZulu-Natal, Pietermaritzburg, South Africa; 18 Central Clinical School, Monash University, Melbourne, Victoria, Australia

**Keywords:** HIV, Reproductive Tract Infections, WOMEN, AFRICA

## Abstract

**ABSTRACT:**

**Objectives:**

In sub-Saharan Africa, approximately 86% of HIV infections in adolescents aged 15–19 years occur among girls. Their heightened susceptibility is likely influenced by converging sociobehavioural and biological factors, although the relative contributions remain unclear. To address this, we compared known and hypothesised risk factors for HIV between cisgender adolescent girls and adult women in South Africa and evaluated the relationships between these factors and sexually transmitted infection (STI) status.

**Methods:**

This cross-sectional observational study included adolescent (n=305; 14–19 years) and adult females (n=114; 25–35 years) in two South African provinces (Western Cape (WC), KwaZulu-Natal (KZN)). Demographic and sociobehavioural data were collected by questionnaire. Colposcopy was conducted to identify cervicovaginal abnormalities, and tests for bacterial vaginosis (BV), *Chlamydia trachomatis*, *Neisseria gonorrhoeae* and *Trichomonas vaginalis* were performed.

**Results:**

Adults reported higher risk sexual behaviour than adolescents across multiple variables, although adolescents were more likely to have STIs than adults (62.8% vs 34.0%, respectively, p=0.0010 for WC; 42.5% vs 16.4%, respectively, p=0.0002 for KZN). Adolescents did, however, report earlier sexual debut (16 years old vs 17 years old, p<0.0001 for both sites) and KZN adolescents were more likely to use intravaginal sexual enhancers than adults (34.6% vs 20.6%, respectively, p=0.0417). Numbers of sexual partners (β-coefficient: 0.34, SE: 0.12, p=0.0054) and sex acts within the previous 3 months (β-coefficient: 0.25, SE: 0.09, p=0.0062) were associated with STIs in adolescents and trended to significance for adults. Intravaginal sexual enhancer use (KZN only; β-coefficient: 0.95, SE: 0.38, p=0.0118) and biological risk factors, including BV Nugent score (β-coefficient: 0.09, SE: 0.04, p=0.0257) and signs of cervicovaginal injury/inflammation (β-coefficient: 1.07, SE: 0.45, p=0.0171), were associated with STIs in adolescents but not adults.

**Conclusions:**

Risk factors for STIs including HIV may differ between age groups of girls and women, and mitigation interventions may need to be tailored accordingly.

**WHAT IS ALREADY KNOWN ON THIS TOPIC**

- Adolescent girls and young women in sub-Saharan Africa are at high risk of HIV acquisition; however, the causes are not fully understood.

**WHAT THIS STUDY ADDS**

- This study helps to address this knowledge gap by identifying differences in known and hypothesised HIV risk factors between adolescent girls (14–19 years) and adult women (25–35 years) in two HIV endemic settings in South Africa. Key differences between age groups included both sociobehavioural factors (sexual risk behaviour, vaginal product use, HIV risk perception and risk-reducing measures) and biological factors (sexually transmitted infection status and signs of cervicovaginal injury or inflammation).

**HOW THIS STUDY MIGHT AFFECT RESEARCH, PRACTICE OR POLICY**

- This study informs HIV prevention strategies, highlighting the importance of tailoring interventions to address social, behavioural and biological risk factors relevant to the needs of specific age groups and populations.

## Introduction

 In sub-Saharan Africa (SSA), approximately six out of seven new HIV infections that occur among adolescents aged 15–19 years are among girls. Young women aged 15–24 years are twice as likely to acquire HIV than their male counterparts.^1^ The causes of this disparate vulnerability are not fully understood and are likely driven by combinations of biological, social and structural factors that differ by age and geographical region.^1^ It is critical to understand why young women in SSA experience such high rates of HIV infection so that interventions can be tailored to reduce risk in this key population.

Biological factors, such as sexually transmitted infections (STIs), non-optimal vaginal microbiota, including bacterial vaginosis (BV), and vulvovaginal candidiasis, increase susceptibility to sexual HIV acquisition and are highly prevalent among women residing in SSA.^2^,^4^ These conditions cause female genital tract (FGT) inflammation which facilitates HIV acquisition by disrupting the epithelial barrier and recruiting HIV target cells.^5^,^7^ In SSA, STIs including *Neisseria gonorrhoeae* (NG), *Chlamydia trachomatis* (CT) and *Trichomonas vaginalis* (TV) are more prevalent among younger (18–24 years) compared with older (25–49 years) women and FGT inflammatory marker concentrations have been found to be higher in younger women after adjusting for STI, but not BV, status.^4 7^ These factors may biologically predispose younger women to HIV acquisition. In contrast, the prevalence of syphilis and herpes simplex virus type 2 is greater in older women, while BV prevalence is similar.^4^ The anatomical state of the FGT also differs between adolescent and adult women, with adolescents more likely to have immature cervicovaginal epithelium or cervical ectopy that could increase the risk of STIs, including HIV.^8^

Sociobehavioural factors that may increase the risk of HIV infection include socioeconomic vulnerability, transactional relationships, gender-based violence (GBV) and vaginal insertion product (VIP) use.^1 9^ Additionally, many young women in SSA engage in relationships with older men which may increase their risk of sexual HIV acquisition due to increased likelihood of the male partner living with HIV, and reduced ability of the woman to negotiate safe sex practices.^10 11^ Intimate partner violence due to gender inequality, unemployment, poverty and alcohol abuse may also increase HIV risk.^12^ We further speculate that sexual activity in adolescent girls may cause more mucosal trauma compared with older women.^13^ In some South African communities, women have reported feeling pressured to please their partners with tight and dry vaginas and thus insert different products for this purpose.^9^ While little is known about the mucosal effects of most VIPs, some products like vinegar, bicarbonate of soda and lemon juice have been found to potentially impact the microbiome and induce vaginal inflammation.^14^,^16^ This study aimed to characterise biological and sociobehavioural factors in adolescent and adult women residing in high-risk areas in South Africa to understand differences that may explain the high incidence of HIV in adolescent girls and young women.

## Methods

### Study cohort

This cross-sectional study describes baseline data collected during the Mucosal Injury from Sexual Contact (MISC) study conducted between 2017 and 2021. MISC was a prospective, longitudinal, observational study that enrolled adolescent girls (14–19 years) and adult women (25–35 years) at two sites in South Africa: (1) the rural Vulindlela Clinical Research Site at the Centre for the AIDS Programme of Research in South Africa in KwaZulu-Natal (KZN), and (2) the periurban Desmond Tutu Health Foundation Adolescent Clinic in Philippi East, Western Cape (WC). Inclusion criteria included: not being pregnant, not having taken antibiotics in the past month, no cervical disease history, HIV negative and prior sexual activity (online supplemental material). Participants were asked to refrain from sex or inserting vaginal products for 2 weeks prior to their baseline visit.

Written informed consent was obtained from participants ≥16 years of age, with a waiver of parental consent from the ethics committees for 16–17 year-olds due to the sensitive nature of the study. For girls between the ages of 14 and 15 years, both assent and parental consent were obtained.

### Demographic, sociobehavioural and clinical data collection

Interviewer-administered structured questionnaires were used to collect information about demographics, sexual behaviour, GBV, vaginal hygiene and sexual enhancement practices, risk perception, income sources, drug and alcohol use, contraceptive use, mental health and parity. Participants were tested for STIs, BV and fungal infections as described in the online supplemental material. An Eva System colposcope (MobileODT, Israel) was used to collect images of the cervix and vagina that were then examined by the study gynaecologist to identify the presence of cervicovaginal abnormalities.

### Statistical analysis

Statistical analyses were conducted using GraphPad Prism V.9.4.0 and RStudio V.1.2.1335. Proportions were compared between adolescents and adults using Fisher’s exact test and continuous and ordinal data using Mann-Whitney U test. Continuous variables were log_10_ transformed and logistic regression used to evaluate associations between each variable and prevalent STIs. Variables with p≤0.1 were included in multivariable logistic regression model to determine the most important factors associated with prevalent STIs.

## Results

### Social characteristics

This study enrolled 305 adolescent girls (14–19 years) and 114 adult women (25–35 years; 25–29 years, n=79, and 30–35 years, n=35) from periurban (Philippi, Cape Town, WC; n=163) and rural (Vulindlela, KZN; n=256) communities in South Africa. The majority of adolescents were high school students, while most adults were unemployed, with their main sources of income being family support or social grants (table 1; online supplemental table 1). Across both sites and age categories, mothers were the most commonly reported heads of households (online supplemental table 1).

**Table 1:** Social, behavioural and clinical characteristics of South African adolescents and adults

	WC			KZN		
	Adolescents% (n/N)	Adults% (n/N)	P value	Adolescents% (n/N)	Adults% (n/N)	P value
Relationship status						
Married	0.0 (0/111)	2.1 (1/48)	0.3019	0.0 (0/191)	1.6 (1/64)	0.2510
Living with partner	**0.0(0/111)**	**14.6(7/48)**	**0.0002**	1.6 (3/191)	6.3 (4/64)	0.0687
Have a partner but living separately	**100(111/111)**	**83.3(40/48)**	**<0.0001**	**97.9(187/191)**	**92.2(59/64)**	**0.0466**
Not in a relationship	0.0 (0/111)	0.0 (0/48)	–	0.5 (1/191)	0.0 (0/64)	>0.9999
Sexual activity						
Age at sexual debut (median (range))	**16 (13–18)**	**17 (13–22)**	**<0.0001**	**16 (8–18)**	**17 (13–23)**	**<0.0001**
Lifetime number of sexual partners (median (range))	**3 (1–10)**	**5 (2–15)**	**<0.0001**	**2 (1–6)**	**3 (1–23)**	**<0.0001**
Largest partner age difference (median (range))	**3 (0–11)**	**7 (0–24)**	**<0.0001**	**3 (−2to 24)**	**5 (0–22)**	**<0.0001**
Condom use at last sex act	54.0 (61/113)	37.5 (18/48)	0.0604	11.0 (20/181)	21.3 (13/61)	0.0528
Number of partners in the last 3 months (median (range))	**1 (1–2)**	**1 (1–3)**	**0.0006**	1 (0–2)	1 (0–2)	0.6917
Number of sex acts in the last month (median (range))	**3 (0–6**)	**5 (1–30)**	**<0.0001**	1 (0–11)	2 (0–15)	0.2450
Ever had anal sex	0.9 (1/112)	2.0 (1/50)	0.5207	**3.7(7/188)**	**14.3(9/63)**	**0.0060**
Ever had transactional sex	**2.7(3/113)**	**26.0(13/50)**	**<0.0001**	**4.3(8/188)**	**20.6(13/63)**	**0.0002**
HIV risk perception						
Low	44.6 (50/112)	44.9 (22/49)	>0.9999	**36.9(69/187)**	**55.6(35/63)**	**0.0119**
Some	52.7 (59/112)	42.9 (21/49)	0.3048	24.0 (45/187)	30.2 (19/63)	0.4040
High	**2.7(3/112)**	**12.2(6/49)**	**0.0237**	**39.0(73/187)**	**14.3(9/63)**	**0.0003**
Practices to protect against HIV						
Nothing	38.1 (43/113)	48.0 (24/50)	0.3004	**83.0(156/188)**	**50.8(32/63)**	**<0.0001**
Male condom	58.4 (66/113)	52.0 (26/50)	0.4952	14.4 (27/188)	25.4 (16/63)	0.0537
Female condom	0.0 (0/113)	0.0 (0/50)	–	0.0 (0/188)	1.6 (1/63)	0.2510
PrEP	2.7 (3/113)	0.0 (0/50)	0.5535	**2.1(4/188)**	**30.2(19/63)**	**<0.0001**
Gender-based violence						
Felt pressure to have sex	2.7 (3/113)	4.0 (2/50)	0.6428	10.1 (19/188)	15.9 (10/63)	0.2545
Pressured to send naked pictures	4.4 (5/113)	4.0 (2/50)	>0.9999	1.6 (3/188)	1.6 (1/63)	>0.9999
Experienced unwanted sexual touching	4.4 (5/113)	2.0 (1/49)	0.6688	1.6 (3/188)	3.2 (2/63)	0.6019
Ever raped	**1.8(2/113)**	**10.0(5/49)**	**0.0270**	**2.1(4/188)**	**8.0(5/63)**	**0.0466**
Ever sexually assaulted	2.7 (3/113)	10.0 (5/49)	0.0552	**2.1(4/188)**	**8.0(5/63)**	**0.0466**
Depression score (median (range))	4 (0–22)	3 (0–8)	0.6192	0 (0–10)	0 (0–16)	0.1209
Intravaginal cleansing	0.0 (0/113)	0.0 (0/50)	–	32.8 (63/192)	28.1 (18/64)	0.5371
Sexual enhancer use	0.0 (0/113)	4.0 (2/50)	0.0928	66.5 (125/188)	69.8 (44/63)	0.6456
Body mass index (median (range))	**23.6 (14.1–55.7)**	**31.9 (17.9–43.4)**	**<0.0001**	**23.7 (16.5–40.4)**	**32.2 (19.1–49.0)**	**<0.0001**
Sexually transmitted infections (STIs)						
Any STI	**62.8(64/102)**	**34.0(17/50)**	**0.0010**	**42.5(65/153)**	**16.4(10/61)**	**0.0002**
*Trichomonas vaginalis*	9.5 (10/105)	12.0 (6/50)	0.7782	7.1 (11/155)	6.5 (4/62)	>0.9999
*Chlamydia trachomatis*	**55.9(57/102)**	**24.0(12/50)**	**0.0002**	**33.4(60/177)**	**9.7(6/62)**	**0.0001**
*Neisseria gonorrhoeae*	15.2 (16/105)	4.0 (2/50)	0.0582	7.9 (14/177)	3.3 (2/61)	0.3721
Bacterial vaginosis	45.5 (45/99)	45.8 (22/48)	>0.9999	38.9 (68/175)	40.3 (25/62)	0.8802
Intermediate bacterial vaginosis	15.1 (15/99)	10.4 (5/48)	0.6088	25.7 (45/175)	17.7 (11/62)	0.2275
Vaginal pH (median (range))	4.7 (3.6–6.1)	5.0 (3.5–5.6)	0.2650	4.7 (3.6–6.1)	4.7 (3.6–5.6)	0.7119
Yeast/fungal hyphae	8.1 (8/99)	8.3 (4/48)	>0.9999	8.2 (14/171)	6.5 (4/62)	0.7867
Colposcopy findings (n=297)						
Any injury/inflammation-related sign (erythema, petechiae, ecchymosis, oedema)	19.4 (14/72)	31.4 (11/35)	0.2235	**10.0(16/160)**	**31.0(18/58)**	**0.0005**
Leukoplakia	25.0 (18/72)	31.4 (11/35)	0.4952	43.8 (70/160)	32.8 (19/58)	0.1625
Cervical ectopy	12.5 (9/72)	17.1 (6/35)	0.5593	12.5 (20/160)	24.14 (14/58)	0.0553
Any visible discharge	**40.3(29/72)**	**20.0(7/35)**	**0.0496**	**29.4(47/160)**	**14.6(8/58)**	**0.0216**
Genital warts	0.0 (0/72)	0.0 (0/35)	–	1.9 (3/160)	0.0 (0/58)	0.5668

Statistically significant differences (p<0.05) are shown in bold.

KZN, KwaZulu-Natal; PrEP, pre-exposure prophylaxis; WC, Western Cape.

### Sexual behaviour and GBV

Reported age of sexual debut was lower in adolescents compared with adults at both sites (16 years vs 17 years for adolescents and adults, respectively, at both sites, p<0.0001; table 1). Anal sex was more commonly reported by KZN adults compared with adolescents (14.3% vs 3.7%, p=0.0060). In WC, adults reported a higher number of sex acts in the previous month than adolescents (5 vs 3; p<0.0001). Adults reported a higher median age difference with a sexual partner than adolescents (p<0.0001 for both sites; table 1). Most participants reported being in stable relationships; however, WC adults had a significantly greater number of casual partners (10.0% vs 1.8%, p=0.0287) and multiple partners (20.0% vs 5.3%, p=0.0078) than adolescents (online supplemental table 1).

In KZN, more adolescents (83.0%) than adults (50.8%) reported using no protection against sexual HIV acquisition (p<0.0001). The low uptake of HIV prevention options may be mediated through risk perception. Large proportions of participants in both age groups and sites perceived their HIV risk as low (44.6% vs 44.9% for WC adolescents and adults, and 36.9% vs 55.6% for KZN adolescents and adults, respectively; table 1). At the WC site, a greater proportion of adults reported perceived high risk compared with adolescents (12.2% vs 2.7%, p=0.0237), while a greater proportion of KZN adolescents reported perceived high risk compared with adults (39.0% vs 14.3%, p=0.0003). Adult women at both sites were more likely to have been previously raped compared with adolescent women (10.0% vs 1.8% and 8.0% vs 2.1% for adults vs adolescents in WC and KZN, respectively; table 1).

### Sexual enhancers and vaginal hygiene practices

**Figure 1:**
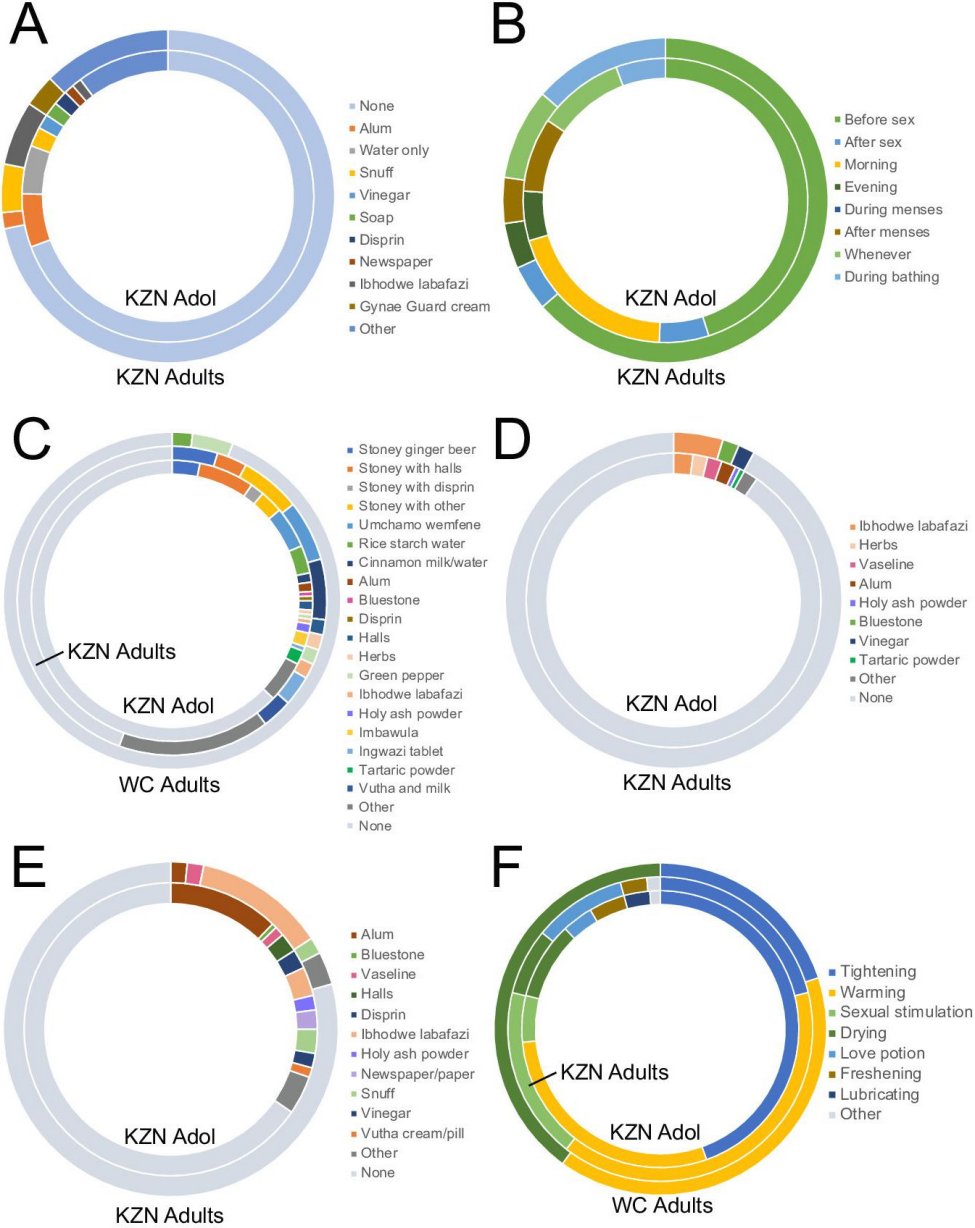
Vaginal hygiene practices and sexual enhancer use by South African adolescents and adults. Information was collected using interviewer-administered questionnaires. (**A**) Proportions of adolescents (Adol) and adults in KwaZulu-Natal (KZN) inserting different substances intravaginally for hygiene purposes (n=256). (**B**) Time of intravaginal hygiene practices. (**C**) Proportions of adolescents (n=188) and adults (n=63) in KZN and adults (n=50) in the Western Cape (WC) ingesting products for sexual enhancement. (**D**) Proportions of adolescents and adults in KZN using vaginal sexual enhancers externally for sexual enhancement. (**E**) Proportions of adolescents and adults in KZN using vaginal sexual enhancers internally for sexual enhancement. (**F**) Reported purpose of using sexual enhancers.

While both adolescents and adults at the WC site reported extravaginal washing with only soap and water, KZN adolescents and adults reported washing internally with a variety of products (table 1; figure 1). Although differences were noted in the products of choice (figure 1A), only the proportions of participants using ‘ibhodwe labafazi’ (scented petroleum jelly with unknown ingredients) for hygiene purposes differed significantly between adolescents compared with adults (4% vs 24%, respectively; p=0.0225). Internal vaginal washing was mostly done before sex in both age groups, indicating that this practice was mainly to prepare for sex (figure 1B).

The majority of KZN adolescents (66.5%) and adults (69.8%) reported using products thought to enhance sexual pleasure (online supplemental table 2). KZN adults were more likely to ingest these products (p=0.0123), whereas adolescents were more likely to use enhancers intravaginally (p=0.0417). The most commonly ingested products were Stoney (soft drink) combined with either Halls (mentholated lozenges) or Disprin (aspirin tablet), and ‘umchamo wemfene’ (baboon’s urine; figure 1C). The most common product applied intravaginally among adolescents was ‘alum’ (aluminium sulfate and potassium sulfate), and among adults were ‘ibhodwe labafazi’ and ‘snuff’ (dried tobacco leaves; figure 1D). Fewer women applied sexual enhancers externally, and the products used were similar to those applied internally (figure 1E; online supplemental table 2). The main reasons for using sexual enhancers were to tighten or warm the vagina (figure 1F). At the WC site, 2/50 adults reported ingesting products (rice water or green pepper; figure 1C) for vaginal warming, drying and tightening (figure 1F).

### Biological and clinical factors

**Figure 2:**
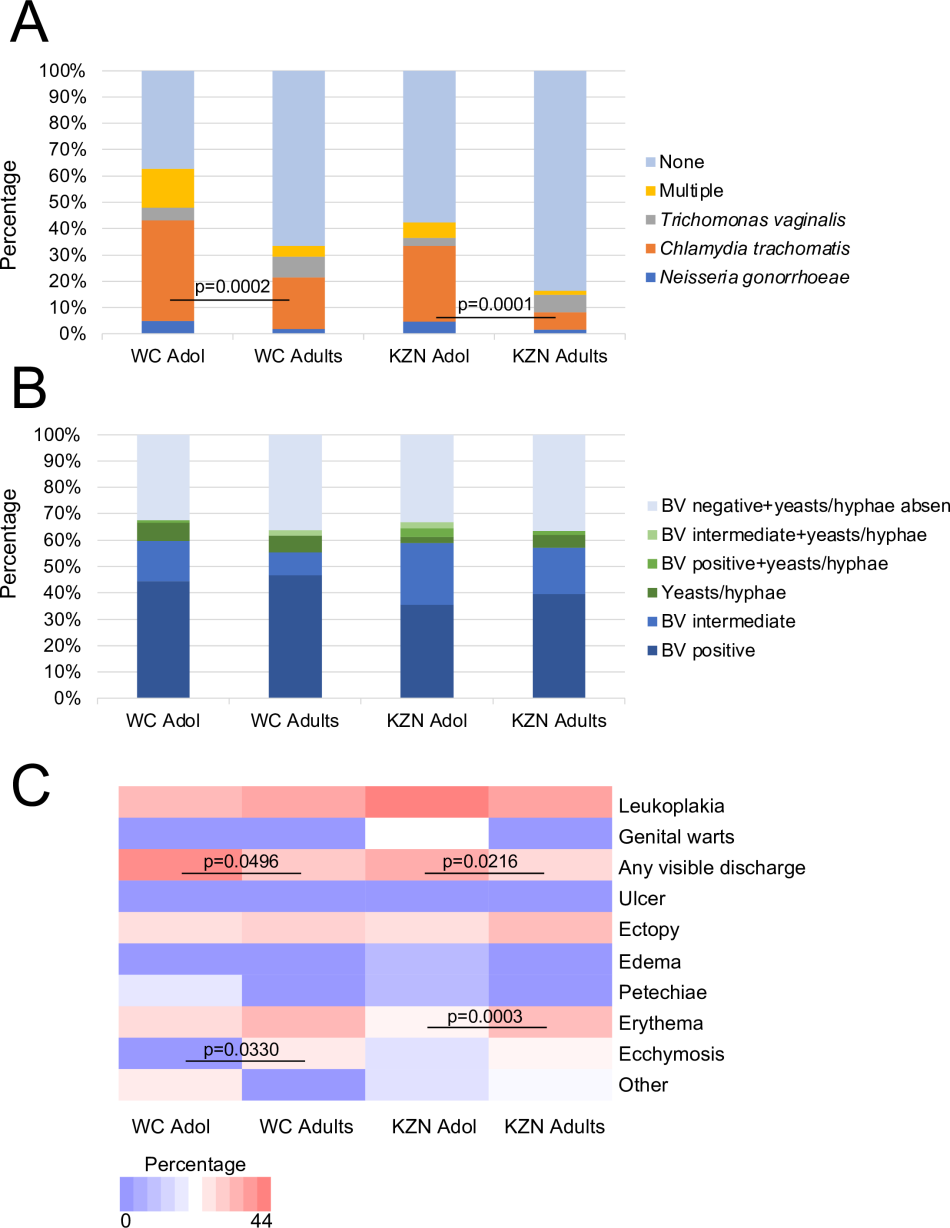
Sexually transmitted infections (STIs), bacterial vaginosis (BV), candidiasis (yeasts/hyphae) and cervicovaginal abnormalities in South African adolescents and adults. (**A**) Women were tested for *Chlamydia trachomatis* and *Neisseria gonorrhoeae* using the Xpert CT/NG assay, and for *Trichomonas vaginalis* using wet mount microscopy and the OSOM Trichomonas Rapid Test in KwaZulu-Natal (KZN). Women in the Western Cape (WC) were tested for these STIs using Primerdesign genesig kits. (**B**) BV was diagnosed using Nugent scoring. The presence of yeast and fungal hyphae was evaluated using a microscopy potassium hydroxide mount in KZN and examination of Gram-stained vaginal smears in WC. (**C**) Colposcopic images of the cervix and vagina were collected by the study nurses and then examined by the study gynaecologist to identify the presence of cervical or vaginal abnormalities. Participants were excluded from analysis if either or both vaginal and cervical images were not collected or blurred, with 325 women included. The heatmap shows the percentage of women with each abnormality. Proportions were compared using Fisher’s exact test and p values <0.05 were considered significant. CT, *Chlamydia trachomatis*; NG, *Neisseria gonorrhoeae*

The prevalence of STIs and BV was high, particularly in WC, with CT significantly more prevalent in adolescents compared with adults at both sites (p=0.0002 and p=0.0001 for WC and KZN, respectively; table 1; figure 2). BV status, the presence of yeast/fungal hyphae and vaginal pH did not differ significantly between adolescents and adults (figure 2). Colposcopy imaging in a subset of participants (n=325) showed that adolescents were more likely to have cervicovaginal discharge compared with adults (p=0.0496 and p=0.0216 for WC and KZN, respectively; table 1). However, adults were more likely to have evident cervicovaginal injury-related or inflammation-related signs compared with adolescents, including ecchymosis (discolouration/bruising) in WC (p=0.0330) and erythema (superficial reddening) in KZN (p=0.0003; figure 2). Cervical ectopy did not differ significantly between age groups.

### Factors associated with prevalent STIs

We next investigated whether sociobehavioural and clinical factors associated with STI status (CT, NG, TV) differed between adolescents and adults. Several sociobehavioural factors positively associated with STIs were similar between age groups, including the WC clinical site (p=0.0069 and p=0.0277 for adolescents and adults, respectively) and experience of deaths in the household (p=0.0375 and p=0.0378 for adolescents and adults, respectively). Reporting of condom use, greater numbers of sexual partners and sexual acts in the previous 3 months tended to be positively associated with prevalent STIs in both groups (table 2). A higher level of alcohol use was a risk factor for STIs in adolescents (p=0.0267), but not adults. In adolescents, several clinical factors were also associated with STIs but not adults, although this may be due to the difference between the sample sizes of the two groups. These included having higher body mass index (BMI; p=0.0250), higher BV Nugent score (p=0.0257), clinical signs of cervicovaginal injury or inflammation (p=0.0171) and visible cervicovaginal discharge (p=0.0223; table 2).

**Table 2:** Characteristics associated with sexually transmitted infection status among South African adolescents and adults

	Adolescents (n=232)			Adults (n=98)		
	β-coefficient	SE	P value	β-coefficient	SE	P value
Demographic and social						
Site	**0.74**	**0.27**	**0.0069**	**1.03**	**0.47**	**0.0277**
Age	0.12	0.11	0.2650	−0.10	0.09	0.2380
Level of education	0.15	0.32	0.6340	0.45	0.67	0.5050
Employment	0.08	0.11	0.4380	0.17	0.26	0.5000
Relationship status	−0.11	0.46	0.8120	−0.02	0.69	0.9720
Deaths in the household	**0.48**	**0.23**	**0.0375**	**0.71**	**0.34**	**0.0378**
Clinical						
BMI	**3.15**	**1.41**	**0.0250**	−1.22	2.49	0.6240
Vaginal pH	0.28	0.22	0.1910	0.46	0.44	0.3010
BV Nugent score	**0.09**	**0.04**	**0.0257**	0.06	0.07	0.3451
Candida	0.24	0.52	0.6430	1.03	0.85	0.2270
Depression (PHQ-9 score)	−0.01	0.04	0.8160	0.08	0.08	0.2890
Colposcopy (n=192)						
Injury/inflammation (erythema, petechiae, ecchymosis, oedema)	**1.07**	**0.45**	**0.0171**	−0.48	0.55	0.3859
Leukoplakia	−0.30	0.30	0.3110	0.54	0.51	0.2907
Ectopy	0.18	0.46	0.7020	0.41	0.58	0.4796
Visible discharge	**0.72**	**0.31**	**0.0223**	−1.64	1.08	0.1276
Behavioural						
Previous pregnancy	0.04	0.30	0.9050	0.45	0.67	0.5053
Intravaginal product use for hygiene	−0.21	0.35	0.5450	0.41	0.47	0.3927
Intravaginal sexual enhancer use	0.44	0.34	0.2030	−16.82	1097.25	0.9878
Anal sex	1.67	1.10	0.1300	0.12	0.73	0.8720
Age at debut	−0.06	0.11	0.5710	0.07	0.11	0.5220
Lifetime number of partners	**0.34**	**0.12**	**0.0054**	0.01	0.06	0.8247
Largest partner age difference	0.07	0.06	0.2190	0.00	0.04	0.9964
Transactional sex	−1.27	0.81	0.1180	0.17	0.52	0.7503
Condom at last sex act	**0.58**	**0.31**	**0.0566**	**0.84**	**0.48**	**0.0821**
Number of sexual partners in previous 3 months	**1.11**	**0.67**	**0.0957**	**1.04**	**0.57**	**0.0698**
Number of vaginal sex acts in previous 3 months	**0.25**	**0.09**	**0.0062**	**0.14**	**0.08**	**0.0804**
Sex under the influence of alcohol	0.20	0.29	0.4810	0.45	0.46	0.3235
Sex during menstruation	0.70	0.49	0.1490	0.31	0.60	0.6060
Risk perception	−0.01	0.17	0.9730	0.03	0.32	0.9240
Use male condoms to protect against HIV	**0.70**	**0.29**	**0.0163**	0.64	0.46	0.1650
Use PrEP to protect against HIV	−0.39	0.92	0.6740	−0.86	0.68	0.2012
Level of alcohol use	**0.27**	**0.12**	**0.0267**	0.02	0.20	0.9211
Level of drug use	0.33	0.24	0.1790	0.50	0.32	0.1130
Contraception						
Nur-Isterate	0.31	0.30	0.3060	−0.69	1.12	0.5360
Male condoms	0.03	0.72	0.9700	0.49	0.77	0.5270
Depo	0.24	0.29	0.3970	−0.59	0.46	0.2060
Implanon	−0.36	0.51	0.4860	0.04	0.64	0.9470
Oral contraceptives	0.03	0.59	0.9630	−14.63	1455.40	0.9920
Gender-based violence						
Previously raped	−1.39	1.13	0.2180	1.05	0.75	0.1580
Previously sexually assaulted	−0.39	0.92	0.6740	0.72	0.80	0.3660

Statistically significant differences (p<0.05) are shown in bold.

BMI, body mass index; BV, bacterial vaginosis; PHQ-9, Patient Health Questionnaire-9; PrEP, pre-exposure prophylaxis.

Multivariable analysis was conducted to determine the strongest predictors of STI status (online supplemental table 3). For adolescents, history of household deaths and BV Nugent score were significantly associated with STI status. In a separate model including colposcopic findings, household deaths, cervicovaginal injury/inflammation-related signs and visible cervicovaginal discharge were significantly associated with STIs (online supplemental table 3). For adults, none of the variables included were significantly associated with STI status (online supplemental table 3). When adolescents were stratified by clinical site, similar trends were seen in both WC and KZN. However, for KZN adolescents, intravaginal sexual enhancer use was associated with an increased likelihood of having an STI (β-coefficient: 0.95, SE: 0.38; p=0.0118).

## Discussion

We characterised known and hypothesised HIV risk factors in adolescent girls and adult women in two HIV endemic settings in South Africa to improve our understanding of factors underlying the high rates of sexual HIV acquisition in adolescent girls and young women. While previous studies have investigated sociobehavioural or biological factors separately,^2 7 17 18^ a strength of this study was the characterisation and integration of both. Adults reported higher risk sexual behaviour than adolescents, including larger partner age differences, higher rates of transactional sex, more sexual partners and sex acts, multiple partners, casual relationships, sex during menstruation and anal sex, each of which has been associated with HIV risk previously.^10^,^1219 20^ The only reported HIV risk factors that were more prevalent in adolescents were increased usage of potentially inflammatory intravaginal sexual enhancers among KZN adolescents and earlier sexual debut. These differences may however be due to recall and/or reporting bias, with possible under-reporting of sexually risky behaviour by adolescents in particular, as well as incorrect perceptions of partner age. Additionally, although the partners of adult women may be more likely to be living with HIV than those of adolescent girls, an equivalent partner age difference may result in a greater power imbalance for adolescent girls. Regardless, many adult women may be dependent on their partners for economic support and living in patriarchal communities and power imbalance may thus be important across the lifespan for women. While we had hypothesised that adolescents may be more likely to experience cervicovaginal mucosal trauma, given the relatively naïve state of the vagina around the time of sexual debut, a greater proportion of adults had cervicovaginal injury or inflammation-related signs. This may be due to more frequent sexual activity reported by adults, or differences in wound healing rates between age groups.^21 22^

Despite greater reported sexual risk behaviour in adults overall, adolescents were 1.8-fold and 2.6-fold more likely to have an STI than adults in WC and KZN, respectively. This could relate to a number of factors including differences in sexual networks or treatment-seeking behaviours between adolescents and adult women and their partners. Additionally, as it has been hypothesised that women are able to develop immunity to CT,^23^ adults in this study, due to longer periods of time since sexual debut, were more likely to have previously been exposed to and developed immunity against CT. Thus, the differences in STI prevalence between adults and adolescents may be explained by other factors in addition to differing risk. A limitation of this study is that different diagnostic tests were used for STIs and fungal infections at each site and thus prevalence could not be compared between sites.

While similar sociobehavioural factors were associated with STIs in both adolescents and adults, biological factors (BV, BMI, cervicovaginal injury/inflammation-related signs, vaginal discharge) were more closely linked to STIs in adolescents. Notably, this relationship was not observed in adults, even though adults were more likely to have cervicovaginal injury/inflammation-related signs. However, this analysis has limitations, including the differing sample sizes between the adolescent and adult groups, with the larger number of adolescents increasing the statistical power to detect significant associations. Additionally, due to the cross-sectional study design, it is not possible to define cause and effect, and injury/inflammation-related signs and visible vaginal discharge may either have been caused by the STIs or may be risk factors for STIs. Our study inclusion/exclusion criteria, such as exclusion of girls and women living with HIV and the requirement for participants to already be sexually active, may have impacted the age groups differently and thus introduced bias. Although we hypothesised that adolescents would be more likely to have cervical ectopy, no significant differences were found between adults and adolescents and ectopy was not associated with STIs. This may be because other factors influence the degree of ectopy, in addition to puberty, including oral contraceptive use, pregnancy and frequency of sexual activity.^8^

Many participants perceived themselves as being at low risk of HIV infection. This perceived low risk may influence the personal importance of risk-reducing behaviours and may explain why most participants reported doing nothing to reduce their risk of HIV. Although a larger proportion of adolescents in KZN reported a high perceived risk of HIV infection, they were also less likely to take measures to protect themselves against HIV infection compared with adults. However, a limitation of this comparison is that the KZN site was concurrently conducting a pre-exposure prophylaxis (PrEP) implementation study, with women able to coenrol, and this may have influenced the degree of PrEP use. In WC, a similar disconnect was observed, but it was adult women who reported higher risk perception, with lower rates of risk-reducing behaviours compared with adolescents. These findings suggest a disconnect between HIV risk perception and risk-reducing behaviour, highlighting a potential intention–action gap, as well as limited female-led prevention options available in this population.

VIP use is highly prevalent in many regions of SSA, particularly KZN, with some products implicated in increased HIV acquisition risk.^24 25^ VIP use may also reduce the integrity of latex condoms^26^ or alter safe sex practices, as young women in KZN have reported that condoms reduced the effectiveness of sexual pleasure-enhancing substances.^27^ Although WC adolescents and adults reported mainly washing externally with water and soap, KZN participants reported washing internally with water and a variety of substances, and using various products to achieve a desired vaginal state in preparation for sex. Adults mainly reported the ingestion of substances believed to have aphrodisiacal effects, whereas adolescents mainly inserted vaginal products believed to have drying and tightening effects, similar to previous reports.^9 28^ Although the effects of many of the VIPs identified in this study are unknown, products like alum (a vaccine adjuvant), vinegar, bicarbonate of soda and lemon juice may alter the vaginal microbiome, increase vaginal inflammatory responses and influence risk of HIV acquisition.^14^,^1625^ Indeed, the use of VIPs by KZN adolescents was associated with increased likelihood of having an STI.

In conclusion, significant differences were observed in reported social and behavioural factors, as well as biological factors, that may influence the risk of HIV and other STIs in adults and adolescents residing in different settings in South Africa. Although there has been significant effort to develop HIV prevention interventions relevant to this population,^29 30^ these findings suggest that continued efforts are needed to understand perceived risk and factors that influence decisions to engage in high-risk behaviours. Additionally, more female-led HIV prevention options are needed and the barriers to accessing and using HIV prevention options need to be addressed through health systems strengthening, health service integration and human-centred design. Interventions should be tailored to address social factors, behaviours and biological risk factors relevant to the needs of specific age groups and populations.

## Supplementary material

10.1136/sextrans-2024-056260 online supplemental material 1

## Data Availability

Data are available upon reasonable request.

## References

[1] UNAIDS Full report - in danger: unaids global aids update 2022. joint united nations programme on hiv/aids 2022; (cc by-nc-sa 3.0 igo).

[2] Gosmann C, Anahtar MN, Handley SA (2017). Lactobacillus-Deficient Cervicovaginal Bacterial Communities Are Associated with Increased HIV Acquisition in Young South African Women. Immunity.

[3] Johnson LF, Dorrington RE, Bradshaw D (2012). The role of sexually transmitted infections in the evolution of the South African HIV epidemic. Tropical Med Int Health.

[4] Torrone EA, Morrison CS, Chen P-L (2018). Prevalence of sexually transmitted infections and bacterial vaginosis among women in sub-Saharan Africa: An individual participant data meta-analysis of 18 HIV prevention studies. PLoS Med.

[5] Arnold KB, Burgener A, Birse K (2016). Increased levels of inflammatory cytokines in the female reproductive tract are associated with altered expression of proteases, mucosal barrier proteins, and an influx of HIV-susceptible target cells. Mucosal Immunol.

[6] Masson L, Mlisana K, Little F (2014). Defining genital tract cytokine signatures of sexually transmitted infections and bacterial vaginosis in women at high risk of HIV infection: a cross-sectional study. Sex Transm Infect.

[7] Masson L, Passmore J-AS, Liebenberg LJ (2015). Genital inflammation and the risk of HIV acquisition in women. Clin Infect Dis.

[8] Venkatesh KK, Cu-Uvin S (2013). Assessing the relationship between cervical ectopy and HIV susceptibility: implications for HIV prevention in women. Am J Reprod Immunol.

[9] Humphries H, Mehou-Loko C, Phakathi S (2019). “You’ll always stay right”: understanding vaginal products and the motivations for use among adolescent and young women in rural KZN. Cult Health Sex.

[10] Kaiser R, Bunnell R, Hightower A (2011). Factors associated with HIV infection in married or cohabitating couples in Kenya: results from a nationally representative study. PLoS ONE.

[11] Stoner MCD, Nguyen N, Kilburn K (2019). Age-disparate partnerships and incident HIV infection in adolescent girls and young women in rural South Africa. AIDS.

[12] Abdool Karim Q, Baxter C (2016). The dual burden of gender-based violence and HIV in adolescent girls and young women in South Africa. S Afr Med J.

[13] Baker RB, Sommers MS (2008). Relationship of genital injuries and age in adolescent and young adult rape survivors. J Obstet Gynecol Neonatal Nurs.

[14] Oleszycka E, Moran HBT, Tynan GA (2016). IL-1α and inflammasome-independent IL-1β promote neutrophil infiltration following alum vaccination. FEBS J.

[15] Hesham H, Mitchell AJ, Bergerat A (2021). Impact of vaginal douching products on vaginal Lactobacillus, Escherichia coli and epithelial immune responses. Sci Rep.

[16] Anukam KC, Reid G (2009). In vitro evaluation of the viability of vaginal cells (VK2/E6E7) and probiotic Lactobacillus species in lemon juice. Sex Health.

[17] Lewis L, Kharsany ABM, Humphries H (2022). HIV incidence and associated risk factors in adolescent girls and young women in South Africa: A population-based cohort study. PLoS ONE.

[18] Mabaso M, Sokhela Z, Mohlabane N (2018). Determinants of HIV infection among adolescent girls and young women aged 15-24 years in South Africa: a 2012 population-based national household survey. BMC Public Health.

[19] Morhason-Bello IO, Kabakama S, Baisley K (2019). Reported oral and anal sex among adolescents and adults reporting heterosexual sex in sub-Saharan Africa: a systematic review. Reprod Health.

[20] Wira CR, Fahey JV (2008). A new strategy to understand how HIV infects women: identification of a window of vulnerability during the menstrual cycle. AIDS.

[21] Engeland CG, Sabzehei B, Marucha PT (2009). Sex hormones and mucosal wound healing. Brain Behav Immun.

[22] Porter KA, Turpin J, Begg L (2016). Understanding the Intersection of Young Age, Mucosal Injury, and HIV Susceptibility. AIDS Res Hum Retroviruses.

[23] Omori R, Chemaitelly H, Althaus CL (2019). Does infection with *Chlamydia trachomatis* induce long-lasting partial immunity? Insights from mathematical modelling. Sex Transm Infect.

[24] Hull T, Hilber AM, Chersich MF (2011). Prevalence, motivations, and adverse effects of vaginal practices in Africa and Asia: findings from a multicountry household survey. J Womens Health (Larchmt).

[25] Low N, Chersich MF, Schmidlin K (2011). Intravaginal practices, bacterial vaginosis, and HIV infection in women: individual participant data meta-analysis. PLoS Med.

[26] Voeller B, Coulson AH, Bernstein GS (1989). Mineral oil lubricants cause rapid deterioration of latex condoms. Contraception.

[27] Sinethemba VN, Meyer-Weitz A, Shumba K (2022). The use of sexual pleasure enhancing substances among Zulu female hair salon workers in Durban, South Africa. J Soc Dev Afr.

[28] Scorgie F, Kunene B, Smit JA (2009). In search of sexual pleasure and fidelity: vaginal practices in KwaZulu-Natal, South Africa. Cult Health Sex.

[29] Chimbindi N, Birdthistle I, Floyd S (2020). Directed and target focused multi-sectoral adolescent HIV prevention: Insights from implementation of the “DREAMS Partnership” in rural South Africa. J Int AIDS Soc.

[30] Naledi T, Little F, Pike C (2022). Women of Worth: the impact of a cash plus intervention to enhance attendance and reduce sexual health risks for young women in Cape Town, South Africa. J Int AIDS Soc.

