# Supplementary material for: Differences in HIV risk factors between South African adolescents and adult women and their association with sexually transmitted infections
Source: Sex Transm Infect. 2024 Dec 4;101(3):e056260. doi: 10.1136/sextrans-2024-056260 (PMC11999788; doi:10.1136/sextrans-2024-056260)
Supplement: online supplemental material 1 [file sextrans-101-3-s001.pdf]

Supplemental Material

Supplemental Methods:

Study cohort:

This cross-sectional study describes baseline demographic, socio-behavioural and biological data collected during the Mucosal Injury from Sexual Contact (MISC) study. Women and girls attending the CAPRISA Vulindlela Clinic Research Site (CVCRS) in KwaZulu Natal, including those participating in oral pre-exposure prophylaxis studies, or visiting the Desmond Tutu Health Foundation in Philippi Village, Cape Town were informed about the study by clinic fieldworkers and study nurses. Additionally, the study was advertised using flyers and posters at the research sites, through other studies being conducted at schools and youth events. For women and girls interested in participating in the study, the study staff explained the study procedures and inclusion/exclusion criteria in greater detail. If still interested in participating, participants completed informed consent and/or assent with parental consent prior to screening. Inclusion criteria included: being aged between 14-19 years (KZN site), or 16-19 years (WC site), according to the requirements of each site's ethical committee, or 25-35 years; willing and able to provide written informed consent or assent; willing to provide locator data; not relocating within 12 months; willing to complete study procedures; not pregnant; not having taken antibiotics in the past month; no cervical disease history; HIV negative; and prior sexual activity. Exclusion criteria included refusal by a participant and/or parent or legal guardian to participate in the study. If participants were identified as requiring antibiotics for STI treatment, treatment was provided, and they were asked to re-screen for the study 30 days following the last antibiotic dose.

For the analyses described herein, a sample size of 305 adolescent girls and 114 adult women had sufficient statistical power (>0.8) to detect medium to large effect sizes (h>0.3) when comparing two proportions at a significance level of 0.05.

Questionnaire:

Socio-behavioral risk profiles were developed from a review of current evidence on the factors associated with increased risk of HIV infection to develop and refine a structured questionnaire. The tool was then further refined through consultations with experts in the fields of psychology and adolescent research. To ensure reliability of the questionnaire, questions relating to multiple levels affecting an individual's specific socio-behavioral risk profile were included. In addition to the questionnaires, all study participants were given diaries to record sexual behavior, coital frequency and vaginal product use to assist with recall and accurate capturing of sexual behavior. Questionnaires were administered by counsellors and nurses who were fully trained and had substantial experience working with adolescents, in comfortable, private, and non-judgmental settings to foster trust and maintain confidentiality.

STI testing:

Rapid HIV testing with HIV risk reduction counselling was done using Uni-Gold™ Recombigen® HIV-1/2 (Trinity Biotech, Ireland) and Determine™ HIV-1/2 test (Abbott Diagnostics Scarborough, Inc., USA) in KZN or One Step Anti-HIV (1&2) (Intec Products Inc., China) and HIV 1+2 Rapid Test (Wantai BioPharm, China) in WC.

In KZN, urine samples were screened for *Chlamydia trachomatis* (CT) and *Neisseria gonorrhoeae* (NG) using the Xpert® CT/NG assay (Cepheid, USA) at the CAPRISA Vulindlela onsite laboratory. Xpert® sensitivities and specificities in urine are 97.6% and 99.8% (CT) and 95.6% and 99.9% (NG), respectively, compared to Gen-Probe Hologic APTIMA Combo 2 assay and the ProbeTec ET *C. trachomatis* and *N. gonorrhoeae* amplified DNA assays as the reference standards [1]. Accuracy was similar compared to vaginal swabs, with sensitivities and specificities of 98.7% and 99.4% (CT) and 87.3% and 99.6% (NG), respectively [1]. Lateral vaginal wall swab samples were used for *Trichomonas vaginalis* (TV) detection with wet mount microscopy combined with OSOM® *Trichomonas* Rapid Test (Sekisui Diagnostics, USA). This test had a sensitivity of 75% and specificity of 100% when used at the point-of-care in the South African context compared to laboratory-based Anyplex II STI-7 Detection [2]. In WC, vaginal swabs were used to test for CT, NG, and TV using genesig® kits (Primerdesign™ Ltd, UK) at the Bio Analytical Research Corporation (BARC) reference laboratory (Johannesburg, South Africa). Primerdesign™ genesig® kits showed 100% sensitivity and specificity compared to cobas® 4800 CT/NG Test in a validation conducted by BARC (unpublished). Vaginal swabs were applied to pH-Fix 0–14 PT™ strips (Macherey-Nagel, Germany) and smeared onto glass slides for Gram-staining and BV Nugent scoring. To test for the presence of yeast and fungal hyphae, lateral wall swab samples collected in KZN were tested using a microscopy potassium hydroxide (KOH) mount (Neuberg Global Laboratories, South Africa), while Gram-stained vaginal smears collected in WC were examined for the presence of visible spores or hyphae.

Colposcopy:

Participants underwent a speculum examination, during which an Eva System colposcope (MobileODT, Israel) was used by trained nurses to collect images of the cervix and vagina.

Images were examined by the study gynecologist to identify the presence of cervicovaginal abnormalities. Participants were excluded from analysis if either or both vaginal and cervical images were not collected or blurred.

References:

1. Gaydos CA, Van Der Pol B, Jett-Goheen M, Barnes M, Quinn N, Clark C, Daniel GE, Dixon PB, Hook III EW, CT/NG study group. Performance of the Cepheid CT/NG Xpert rapid PCR test for detection of Chlamydia trachomatis and Neisseria gonorrhoeae. Journal of clinical microbiology. 2013 Jun;51(6):1666-72.
2. Garrett N, Mitchev N, Osman F, Naidoo J, Dorward J, Singh R, Ngobese H, Rompalo A, Mlisana K, Mindel A. Diagnostic accuracy of the Xpert CT/NG and OSOM Trichomonas Rapid assays for point-of-care STI testing among young women in South Africa: a cross-sectional study. BMJ open. 2019 Feb 1;9(2):e026888.

Supplemental Table 1. Characteristics of of adolescents and adults residing in two South African provinces, Western Cape and KwaZulu-Natal

	WC			KZN		
	Adolescents % (n/N)	Adults % (n/N)	p-value	Adolescents % (n/N)	Adults % (n/N)	p-value
**Highest education**						
No education	4.5 (5/112)	0.0 (0/50)	0.3250	0.0 (0/192)	0.0 (0/64)	-
Primary school	0.0 (0/112)	0.0 (0/50)	-	1.6 (3/192)	0.0 (0/64)	0.5754
High school	82.1 (92/112)	78.0 (39/50)	0.5251	97.9 (188/192)	93.8 (60/64)	0.1104
Tertiary / vocational	11.6 (13/112)	22.0 (11/50)	0.0974	**1.1 (1/192)**	**6.3 (4/64)**	**0.0147**
**Current employment**						
Student / learner	**77.9 (88/113)**	**14.0 (7/50)**	**<0.0001**	**84.9 (163/192)**	**4.7 (3/64)**	**<0.0001**
Informally employed	0.0 (0/113)	2.0 (1/50)	0.3067	**0.0 (0/192)**	**4.7 (3/64)**	**0.0151**
Formally employed	**0.9 (1/113)**	**10.0 (5/50)**	**0.0108**	**0.0 (0/192)**	**7.8 (5/64)**	**0.0009**
Unemployed	**21.2 (24/113)**	**74.0 (37/50)**	**<0.0001**	**15.1 (29/192)**	**82.8 (53/64)**	**<0.0001**
**Source of income**						
Salary	**0.9 (1/113)**	**12.0 (6/50)**	**0.0036**	5.2 (10/192)	10.9 (7/64)	0.1443
Partner support	**1.8 (2/113)**	**12.0 (6/50)**	**0.0108**	0.5 (1/192)	3.1 (2/64)	0.1551
Social grant	**1.8 (2/113)**	**40.0 (20/50)**	**<0.0001**	**20.8 (40/192)**	**45.3 (29/64)**	**0.0003**
None	**1.8 (2/113)**	**10.0 (5/50)**	**0.0287**	0.0 (0/192)	0.0 (0/64)	-
Family support	**97.3 (110/113)**	**42.0 (21/50)**	**<0.0001**	**90.1 (173/192)**	**65.6 (42/64)**	**<0.0001**
Other	**0.0 (0/113)**	**14.0 (7/50)**	**0.0002**	0.5 (1/192)	1.6 (1/64)	0.4382
**Head of the household**						
Birth father	33.6 (38/113)	22.0 (11/50)	0.1442	20.3 (39/192)	25.0 (16/64)	0.4825
Sibling (>18 years)	4.4 (5/113)	6.0 (3/50)	0.7018	0.5 (1/192)	1.6 (1/64)	0.4382
Birth mother	38.1 (43/113)	36.0 (18/50)	0.8618	33.3 (64/192)	34.4 (22/64)	0.8796
Sibling (<18 years)	0.9 (1/113)	2.0 (1/50)	0.5207	1.0 (2/192)	1.6 (1/64)	>0.9999
Grandparent	**16.8 (19/113)**	**0.0 (0/50)**	**0.0009**	**44.3 (85/192)**	**10.9 (7/64)**	**<0.0001**
Aunt	5.3 (6/113)	6.0 (3/50)	>0.9999	7.8 (15/192)	9.4 (6/64)	0.7926
Me	**0.0 (0/113)**	**20.0 (10/50)**	**<0.0001**	**0.0 (0/192)**	**10.9 (7/64)**	**<0.0001**
Uncle	1.8 (2/113)	0.0 (0/50)	>0.9999	3.7 (7/192)	6.3 (4/64)	0.4748
My partner	**0.0 (0/113)**	**8.0 (4/50)**	**0.0081**	**0.0 (0/192)**	**6.3 (4/64)**	**0.0036**
**Death in household**						
One	25.9 (29/112)	28.6 (14/49)	0.8467	19.3 (37/192)	15.6 (10/64)	0.5801
More than one	**8.9 (10/112)**	**57.1 (28/49)**	**<0.0001**	4.2 (8/192)	1.6 (1/64)	0.4575
**Contraceptive use**	96.5 (109/113)	88.0 (44/50)	0.0696	56.7 (106/187)	71.9 (46/64)	0.0380
Nur-Isterate	**42.5 (48/113)**	**4.0 (2/50)**	**<0.0001**	16.0 (30/187)	9.4 (6/64)	0.2203
Depot medroxyprogesterone acetate	31.0 (35/113)	42.0 (21/50)	0.2109	**27.3 (51/187)**	**48.4 (31/64)**	**0.0031**
Male condom	**3.5 (4/113)**	**16.0 (8/50)**	**0.0085**	3.2 (6/187)	1.6 (1/64)	0.6820
Implanon	15.9 (18/113)	22.0 (11/50)	0.3784	**2.7 (5/187)**	**9.4 (6/64)**	**0.0343**
Oral contraceptives	0.9 (1/113)	0.0 (0/50)	>0.9999	7.0 (13/187)	1.6 (1/64)	0.1255
Traditional method	0.0 (0/113)	0.0 (0/50)	-	0.5 (1/187)	0.0 (0/64)	>0.9999
**Ever pregnant**	**23.0 (26/113)**	**92.0 (46/50)**	**<0.0001**	28.2 (53/188)	88.9 (56/63)	**<0.0001**

**Number of pregnancies [median (range)]**	**1 (1-1)**	**1 (1-3)**	**<0.0001**	**1 (1-2)**	**1.5 (1-4)**	**<0.0001**
**Unplanned pregnancies**	**100.0 (25/25)**	**77.3 (58/75)**	**0.0056**	**1 (0-2)**	**1 (0-4)**	**0.0001**
**Ever had a miscarriage**	**0.9 (1/113)**	**12.0 (6/50)**	**0.0036**	**3.7 (7/187)**	**15.9 (10/63)**	**0.0023**
**Regular menstruation**	72.6 (82/113)	66.0 (33/50)	0.4570	60.1 (113/188)	34.4 (22/64)	0.0005
**Amenorrhoea**	26.5 (30/113)	34.0 (17/50)	0.3528	18.1 (34/188)	30.0 (19/64)	0.0532
**Partners in last 3 months**						
Stable relationship	94.7 (107/113)	86.0 (43/50)	0.1122	91.4 (171/187)	87.3 (55/63)	0.3307
Casual relationship	**1.8 (2/113)**	**10.0 (5/50)**	**0.0287**	4.3 (8/187)	4.8 (3/63)	>0.9999
Married	0.0 (0/113)	4.0 (2/50)	0.0928	0.0 (0/187)	1.6 (1/63)	0.2520
Multiple partners	**5.3 (6/113)**	**20.0 (10/50)**	**0.0078**	5.9 (11/187)	9.5 (6/63)	0.3845
Forced	0.0 (0/113)	0.0 (0/50)	-	1.6 (3/187)	0.0 (0/63)	0.5742
**Sex under the influence of alcohol**	47.8 (54/113)	64.0 (32/50)	0.0629	17.0 (32/188)	23.8 (15/63)	0.2635
**Sex under the influence of drugs**	0.0 (0/113)	0.0 (0/50)	-	0.0 (0/188)	1.6 (1/63)	0.2510
**Sex during mentruation**	17.9 (20/112)	22.0 (11/50)	0.5251	**2.2 (4/185)**	**11.1 (7/63)**	**0.0070**
**Alcohol use**						
Never	23.4 (26/111)	20.0 (10/50)	0.6873	63.8 (120/188)	68.3 (43/63)	0.5462
Once or twice	74.8 (83/111)	80.0 (40/50)	0.5503	28.2 (53/188)	22.2 (14/63)	0.4125
Monthly	0.0 (0/111)	0.0 (0/50)	-	6.4 (12/188)	6.4 (4/63)	>0.9999
Weekly	1.8 (2/111)	0.0 (0/50)	>0.9999	1.6 (3/188)	3.2 (2/63)	0.6019
Daily/Almost Daily	0.0 (0/111)	0.0 (0/50)	-	0.0 (0/188)	0.0 (0/63)	-
**Drug use**						
Marijuana	6.3 (7/111)	8.0 (4/50)	0.7400	3.2 (6/188)	0.0 (0/63)	0.3416
Cocaine	0.0 (0/111)	0.0 (0/50)	-	0.0 (0/188)	0.0 (0/63)	-
Tik/amphetamines	0.0 (0/111)	0.0 (0/50)	-	0.0 (0/188)	0.0 (0/63)	-
Inhalants	0.0 (0/111)	0.0 (0/50)	-	1.06 (2/188)	0.0 (0/63)	>0.9999
Sedatives/sleeping pills	0.0 (0/111)	0.0 (0/50)	-	1.06 (2/188)	0.0 (0/63)	>0.9999
Nyaope Whoonga	0.0 (0/111)	0.0 (0/50)	-	0.0 (0/188)	0.0 (0/63)	-
Opioids	0.0 (0/111)	0.0 (0/50)	-	0.0 (0/188)	0.0 (0/63)	-
Other	1.8 (2/111)	14.0 (7/50)	0.0042	0.5 (1/188)	1.6 (1/62)	0.4352

WC: Western Cape; KZN: KwaZulu Natal

Supplemental Table 2. Vaginal products used for sexual enhancement

Category	Product name	Ingredients	Reported reason for use
Ingested	Stoney ginger beer	Carbonated water, sugar, citric acid, stabilisers, preservatives (sodium benzoate, potassium sorbate), flavouring, non-nutritive sweeteners	For vaginal lubrication and tightness, can be combined with black Halls sweets for a better result
Ingested/internally applied	Halls	Sugar, glucose syrup, menthol, acids, eucalyptus oil, acidity regulators	Enhances sexual pleasure by giving a unique sensation during sexual activity
Ingested/internally applied	Disprin	A tablet or powder with a main ingredient of 500mg aspirin (acetylsalicylic acid)	Sexual enhancer
Ingested	"Umchamo wemfene" (Baboon's urine)	Made from various herbs and plants such as *Helichrysum odoratissimum,* African Wormwood (Artemisia afra), *Piper guineense* and other herbs and plants depending on regional variations and cultural practices. Umchamo Wemfene may also contain additional ingredients wild garlic and ginger	Sexual stimulation, vaginal tightening and love potion
Ingested	Rice starch water	Starchy water left over after rice is cooked or left to soak	To get minerals and probiotics that will boost the libido
Ingested	Cinnamon milk/water	Fresh milk, cinnamon spice	Cinnamon is a sexual stimulant. When combined with milk it is thought to tighten vaginal muscles and increase libido
Ingested/internally or externally applied	Alum	Aluminum potassium sulfate, chemical formula is $KAl(SO_4)_2$	Vaginal cleansing and to tighten vaginal muscles
Ingested/internally or externally applied	Bluestone	Copper sulphate powder	Sexual enhancer
Ingested/externally applied	Various traditional herbs with Zulu names: "Awe ma", "Isivuthevuthe", "Unukani", "Mkhize", "Vutha", "straw"	Naturally occurring, plant-based products with minimal or no chemical processing	To tighten vaginal muscles and love potion
Ingested	Green pepper	Raw green pepper vegetable, high in iron and vitamin C	Stimulates sexual desire, reduces sexual anxiety
Ingested/internally or externally applied	"Ibhodwe labafazi" (Women's pot)	Scented petroleum jelly	Increases energy and sexual desire
Ingested/internally or externally applied	Holy ash powder	Made of dried desi cow dung cakes	Protection from negativity, and gain favour with the partner
Ingested	"Imbawula" (The Fire)	White powder (unknown ingredients) that can be mixed with Stoney ginger beer and taken orally	Sexual stamina, warming of the vagina and love potion

Ingested	Ingwazi Tablets	Effervescent Tablets made from undisclosed traditional herbs	To improve sexual desire in women, fight infections
Ingested/externally applied	Tartaric powder	Tartaric acid is a white, crystalline organic acid that occurs naturally, chemical formula $C_4H_6O_6$	Tightens the vagina, warms the vagina
Ingested/internally applied	"Vutha" pill/cream (Burning pill/ cream)	Pill and cream made from traditional herbs (unknown ingredients)	Keeps the vagina warm and used as a sexual enhancer
Internally applied	Herbal vaseline	Petroleum jelly mixed with herbs	Sexual enhancement and lubrication
Externally applied	"Indlovukazi" Vaseline (Vaseline for the Queen)	Special petroleum jelly with traditional herbs	Sexual enhancement
Internally applied	Bicarbonate of soda and vinegar	Mixture of baking soda (sodium bicarbonate), a chemical compound with the formula $NaHCO_3$), with apple cider vinegar (crushed fermented apples, yeast, and sugar) and diluted in water.	Sexual enhancer and fight infections
Internally applied	Newspaper/paper	Paper	Dries the vagina
Internally applied	Snuff/"Nstu"	Smokeless tobacco, with nicotine	To tighten vaginal muscles, warm the vagina and for sexual stimulation
Ingested	Borstol	Contains a variety of ingredients including ammonium chloride, sucrose 724mg, ethyl alcohol 11% v/v	Sexual enhancement
Ingested	Brown sugar and lemon	Freshly squeezed lemon juice and brown sugar	Sexual enhancement
Ingested	NIP or "iniphu" (50ml of alcohol)	Vodka made from selected grains, but may also be made from other raw materials such as corn or wheat, depending on the region	Tightens and warms the vagina
Ingested	"Umlilo wabafazi" (fire for women)	Made from various herbs and plants - their specific names are unknown	Warms the vagina, love potion, sexual stamina
Ingested	"Khemani" (cry and come closer)	A mix of variety of herbs and plants, undisclosed by the producer	Love potion
Ingested	Amoeba tea (Kombucha tea)	A fermented drink made from tea, sugar, bacteria, and yeast. Made by cultivating a culture of bacteria and yeast. This culture is then mixed with tea and sugar, and the mixture is left to ferment.	Sexual enhancement
Ingested	Ilanga mixed with water (named after a company Ilanga Herbals)	Tonic made from various herbs and plants - their specific names are unknown	Sexual enhancer, warms the vagina

Ingested	"Ubsuku bonke" (all night)	Traditional remedies made of herbs turned into a powder	Tightens the vagina
Internally applied	"Ugogotshitshi/ tsitsi" (virgin-granny)	Water mixed with unknown ingredients	Tightens and dries the vagina. Love potion
Internally applied	Ice cubes	Frozen water	Sexual enhancement, vaginal tightening
Internally applied	"Iphilisi labasha" (a pill for the young people)	Pill made from undisclosed ingredients	Sexual enhancement
Internally applied	"Itshe lomgodi" (a stone for the hole)	Crystalized aluminum potassium sulfate, chemical formula is $KAl(SO_4)_2$	Vaginal cleansing and to tighten vaginal muscles
Internally applied	"Idliso locansi" (love portion through sexual intercourse)	Unspecified oil that is mixed with herbs	Sexual enhancement and love potion
Externally applied application	"Impukane" (a fly-he becomes persistent like a fly)	Oil combined with a herbal mixture	Drying sexual stimulant
Externally applied	"Umxovo" (a mixture)	A variety of products made by women or sold in traditional chemists	Mainly for sexual enhancement
Externally applied	"Mlomomnandi" (tasty lips)	Ointment with undisclosed ingredients	Love potion, sexual enhancement, and power of persuasion
Externally applied	"Umlotha wamamoya" (spiritual ash)	Known as Indian Ash with undisclosed ingredients	Opens luck and removes evil spirits

Supplemental Table 3. Multivariable models including characteristics associated with sexually transmitted infection status among South African adolescents and adults

	Adolescents (n=232)*		
	β-coefficient	SE	P-value
Site	0.02	0.38	0.9560
**Deaths in the household**	**0.55**	**0.25**	**0.0272**
BMI	2.95	1.56	0.0580
**BV Nugent score**	**0.09**	**0.04**	**0.0363**
Lifetime number of partners	0.19	0.14	0.1845
Number of sexual partners in previous 3 months	0.58	0.72	0.4201
Number of vaginal sex acts in previous 3 months	0.16	0.10	0.1209
Use male condoms to protect against HIV	0.54	0.36	0.1372
Level of alcohol use	0.10	0.15	0.4896
	**Adolescents (n=192)#**		
	**β-coefficient**	**SE**	**P-value**
Site	-0.09	0.45	0.8462
**Deaths in the household**	**0.71**	**0.29**	**0.0136**
BMI	3.36	1.90	0.0765
BV Nugent score	0.04	0.05	0.3540
Lifetime number of partners	0.24	0.17	0.1521
Number of sexual partners in previous 3 months	0.87	0.86	0.3104
Number of vaginal sex acts in previous 3 months	0.15	0.11	0.1880
Use male condoms to protect against HIV	0.47	0.44	0.2845
Level of alcohol use	0.12	0.16	0.4596
**Injury/inflammation (erythema. petechiae. ecchymyosis. edema)**	**1.01**	**0.50**	**0.0442**
**Visible discharge**	**0.90**	**0.35**	**0.0106**
	**Adults (n=98)**		
	**β-coefficient**	**SE**	**P-value**
Site	0.44	0.58	0.4400
Deaths in the household	0.41	0.38	0.2744
Condom at last sex act	0.69	0.52	0.1813
Number of sexual partners in previous 3 months	0.63	0.63	0.3185
Number of vaginal sex acts in previous 3 months	0.06	0.09	0.5119

*Excluding colposcopy results; #Including colposcopy results. SE: standard error; BMI: body mass index; BV: bacterial vaginosis
